# Long non-coding RNA TUG1 mediates 5-fluorouracil resistance by acting as a ceRNA of miR-197-3p in colorectal cancer

**DOI:** 10.7150/jca.32065

**Published:** 2019-08-07

**Authors:** Meng Wang, Hanqing Hu, Yuliuming Wang, Quanlong Huang, Rui Huang, Yinggang Chen, Tianyi Ma, Tianyu Qiao, Qian Zhang, Hongyu Wu, Qingmin Chen, Dong Han, Guiyu Wang, Xishan Wang

**Affiliations:** 1 Department of Colorectal Surgery, the Second Affiliated Hospital of Harbin Medical University, Harbin 150081, China; 2 Department of Biochemistry and Molecular Biology, Harbin Medical University, Harbin 150081, China; 3 Department of General Surgery, Hongqi Hospital Affiliated to Mudanjiang Medical University, Mudanjiang 157011, China; 4 Department of Colorectal Surgery, Cancer Institute and Hospital, Chinese Academy of Medical Sciences, Peking Union Medical College, Beijing 100000, China

**Keywords:** Colorectal cancer, TUG1, miR-197-3p, TYMS, ceRNA

## Abstract

One major reason for the failure of advanced colorectal cancer (CRC) treatment is the occurrence of chemoresistance to fluoropyrimidine (Fu)-based chemotherapy. Accumulating evidence indicates that long non-coding RNAs (lncRNAs) play a critical role in cancerous processes as either oncogenes or tumor suppressor genes. Here, we observed lncRNA TUG1 was associated to the 5-Fu resistance in colorectal cancer. Firstly, quantitative analysis indicated that TUG1 was significantly increased in recurrence CRC patient samples. Kaplan-Meier survival analysis indicated that high TUG1 expression in CRC tissues was significantly associated with a higher rate of disease progression. TUG1 knockdown re-sensitized the 5-Fu resistance in colorectal cancer cells, which were 5-Fu-resistant colorectal cell line. Furthermore, bioinformatics analysis showed that miR-197-3p could directly bind to TUG1 suggesting TUG1 might work as a ceRNA to sponge miR-197-3p. Extensively, our study also showed that TYMS was the direct target of miR-197-3p in CRC cells. Taken together, our study suggests that TUG1 mediates 5-Fu resistance in CRC via miR-197-3p/TYMS axis.

## 1. Introduction

Colorectal cancer (CRC) is the third most prevalent cancer type and the third leading cause of cancer-related deaths worldwide 1. The occurrence and progression of CRC is a multi-step process involving in the deregulation of multiple oncogenes and tumor suppressors 2. Combined perioperative chemotherapy and surgery is a major therapeutic treatment for CRC patients 3. Although many novel drugs have been developed for patients with advanced CRC, 5-fluorouracil (5-Fu) is still widely used as the first-line systemic chemotherapy, which targets thymidylate synthase to exert anticancer effects through blocking the normal synthesis of DNA and disrupting RNA processing 4. However, the clinical response to 5-Fu varies greatly; chemotherapy resistance is considered to be a major reason for CRC therapy failure 5. Therefore, it is essential to understand the mechanisms of 5-Fu resistance in cancer cells.

Long noncoding RNAs (lncRNAs) are a class of RNA molecules with a length longer than 200 nucleotides (nts) that do not code for proteins 6. Multiple lines of evidence have shown that lncRNAs participate in various biological processes, including chromosome organization, gene imprinting, gene transcription, and alternative splicing 7. For example, HOTAIR is generally deregulated and can regulate chromatin dynamics and gene expression in several types of cancer, including CRC 8. Our group has showed that long noncoding RNA H19 indicates a poor prognosis of colorectal cancer and promotes tumor growth by recruiting and binding to eIF4A3 9. Previously, we summarized recent progress in the genome-wide analysis of lncRNAs in CRC and the dysregulation of lncRNAs in CRC tissues or cells in a review 10.

Recent researches demonstrate that there is a relationship between the drug-resistance and the aberrantly expressed lncRNAs 11. To date, several lncRNAs were found to mediate drug resistance, for example, Zheng and colleagues found that H19 contributing to cisplatin resistance by regulating glutathione metabolism in ovarian cancer 12. LncRNA HULC attenuates the chemosensitivity of 5-Fu and oxaliplatin by triggering autophagy in HCC cells 13. Among these lncRNAs, Taurine Upregulated Gene 1 (TUG1) was originally identified as a transcript upregulated by taurine, whose function is associated with retinal development 14. In the context of human malignancies, it is overexpressed in bladder cancer, gastric cancer and cervical cancer 15-17, whereas it is downregulated in non-small cell lung cancer 18, suggesting context-dependent roles in different types of cancers. Recently, researchers found that TUG1 mediated methotrexate resistance in colorectal cancer via miR-186/CPEB2 axis 19. However, the functional link between TUG1 and the acquisition of 5-Fu resistance is unclear.

In this study, we explored the altered expression of TUG1 in both 5-Fu-resistant human colon cancer cell lines and clinical patients' samples. We further investigated the biological role of TUG1 in 5-Fu resistant cells. Our results provide experimental evidence that increased TUG1 expression in 5-Fu resistant tumors and mediated 5-Fu resistance via sponging miR-197-3p. Extensively, bioinformatics and experimental analysis revealed that TYMS was the direct and functional target of miR-197-3p in CRC cell chemoresistance. Taken together, we conclude that TUG1 mediates 5-Fu resistance in CRC cells via miR-197-3p/TYMS axis.

## 2. Materials and Methods

### 2.1. Tissue samples and clinical data collection

A total of 124 human CRC tissues and para-tumor tissues were collected in the Department of Colorectal Cancer Surgery (January 2013 to January 2016), the Second Affiliated Hospital of Harbin Medical University. The study was approved by the Research Ethics Committee of Harbin Medical University (Harbin, Heilongjiang, P.R. China), and written informed consent was obtained from all of the patients. The inclusion criteria were: 1) Patients were pathologically and clinically diagnosed with colorectal cancer; 2) After radical surgical debulking, pathology confirmed state Ⅱ or state Ⅲ according to the AJCC cancer staging manual (Seventh Edition), in addition state Ⅱ patients with high-risk characteristics: number of lymph nodes analyzed after surgery (<12), poor prognostic features (poorly differentiated histology; lymphatic/vascular invasion; bowl obstruction; perineural invasion (PNI); localized perforation; close, indeterminate, or positive margins); 3) After surgical debulking, patients had undergone XELOX regimen therapy (Oxaliplatin 130 mg/m^2^ over two hours on the first day; Capecitabine 1000 mg/m^2^, twice daily for 14 days; Repeated every three weeks) or mFOLFOX6 regimen therapy (Oxaliplatin 85mg/m^2^, Leucovorin 400mg/m^2^, 5-Fu 400mg/m^2^ on day 1, then 1200mg/m^2^/day×2 days continuous infusion; Repeat every 2 weeks); 4) Preoperative didn't receive neoadjuvant therapy. The exclusion criteria were: 1) Patients age of 70 or older; 2) Patients couldn't complete at least 3 months adjuvant chemotherapy; 3) Multiple primary cancer; 4) The clinicopathological data were not complete. Recurrence was monitored by imaging examination systems (Chest X-ray and CT), gastrointestinal endoscopy with biopsy, and telephone follow-up. The clinicopathological characteristics of the CRC patients are summarized in Table 1.

### 2.2. Cell culture

The 5-Fu resistant CRC cell line HCT8Fu and its parental sensitive cell line HCT8 were purchased from Shanghai Meixuan Corporation (Shanghai, China). HCT116 and SW1116 cell lines were purchased from the American Type Culture Collection (ATCC, Manassas, VA, USA) and were grown in Dulbecco's modified Eagle's medium (DMEM) or L15 medium (Gibco Laboratories, Grand Island, NY) supplemented with 10% fetal bovine serum (GIBCO, Carlsbad, CA, U.S.A.). HCT8Fu cell line was generated in a stepwise manner by exposing drug-sensitive HCT8 cells to increasing doses of 5-Fu. HCT8 cell line was cultured in Dulbecco's Modified Eagle's medium (GIBCO, Carlsbad, CA, U.S.A.) supplemented with 10% fetal bovine serum (GIBCO, Carlsbad, CA, U.S.A.). HCT8Fu cell line was cultured in RMPI 1640 medium (GIBCO, Carlsbad, CA, U.S.A.) with 10% fetal bovine serum and 15μg/ml 5-Fu (Sigma-Aldrich, Northbrook, IL, U.S.A.) according to the manufacturer's protocols. Both kinds of cells were maintained in an atmosphere containing 5% CO2 at 37℃.

### 2.3. RNA extraction and qRT-PCR analyses

The total RNA was extracted from the tissues or cultured cells using TRIzol reagent (Invitrogen, Carlsbad, CA). Reverse transcribed complementary DNA was synthesized with random primers or microRNAs specific stem-loop primers. 1 μg RNA was reverse transcribed in a final volume of 20 μl by using the PrimeScript RT reagent Kit (TaKaRa, Dalian, China). SYBR Premix Ex Taq (TaKaRa, Dalian, China) were used to examine the expression of TUG1, according to the manual. β-actin and U6 were used as internal controls. Primers sequences were listed in Table S1 and S2. The qPCR results were evaluated and afterwards converted to fold changes.

### 2.4. Cell transfection

Transfections were carried out using lipofectamine 2000 reagent (Invitrogen) according to manufactures instructions. siRNA against TUG1 and a non-targeting siRNA control (Gene Pharma Company, Shanghai, China) were used to knockdown gene expression. MiR-197-3p mimics, NC (negative control) mimics, NC inhibitor and miR-197-3p inhibitor were purchased from Gene Pharma Company (Shanghai, China) (Table S3 and S4).

### 2.5. MTT assays

Exponentially growing cells were seeded at 10,000 cells (100μl culture medium) per well in 96-well plates and incubated for 12 h. The cells were then exposed to different concentrations of 5-Fu, then 20 μl of MTT (Sigma Chemicals, St. Louis, MO, USA; 5 mg/ml in PBS) was added to each well, and the cells were cultured for an additional 4 h. Subsequently, 200 μl of DMSO was added to each well to dissolve the crystals. The values of the optical density at 490 nm were then measured using a micro-plate reader. For TUG1 siRNA transfection, cancer cells were transfected with TUG1 siRNA and then the cell growth were also examined by MTT assay.

### 2.6. Colony formation assay

Cells were plated in 24-well plates at 1×10^5^ cells per well and transfected with si-TUG1 or negative control by using Lipofectamine 2000 (Invitrogen). Then, cells were collected and seeded (300 cells per well) in a fresh six-well plate 48 h after transfection, and maintained in RPMI 1640 containing 10% fetal bovine serum. After 14 days, cells were fixed with 4% paraformaldehyde for 10 min and then stained using crystal violet. Individual colonies (>50 cells) were manually counted.

### 2.7. Flow cytometric analysis

Colon cancer cells treated with 5-Fu, 5-Fu plus si-TUG1 or 5-Fu plus miR-197 mimics for flow cytometry analysis using an Annexin V Apoptosis Detection Kit (Becton Dickinson, NJ, USA), untreated group was considered as control. Cells were stained with Annexin V-fluorescein isothiocyanate (FITC), Propidium Iodide (PI) for 25 min, and then analyzed by flow cytometry (BD CantoII). FACS data were analyzed using FlowJo (Tree Star, Inc.).

### 2.8. Western blot assay and antibodies

Cellular protein extracts were separated in a 12 or 8% SDS-polyacrylamide gel and electrophoretically transferred onto a PDVF membrane (Millipore, Bedford, MA, USA). Membranes were blocked overnight with 5% non-fat dried milk and incubated with antibodies to TYMS (Abcam Biotechnology, USA, 1:1000) or β-actin (Santa Cruz Biotechnology, Santa Cruz, CA, USA, 1:1000) overnight at 4°C. After washing with PBST, the membranes were incubated with horseradish peroxidase-linked secondary antibody. The proteins were visualized using ECL chemiluminescence. Bands were quantified with Image J (National Institutes of Health, Bethesda, MD, USA).

### 2.9. Luciferase reporter assay

The DNA oligonucleotide and the pMiR-Reporter Vector were used to build the luciferase report vectors (pMiR-TUG1-WT/pMiR-TUG1-Mut and pMiR-TYMS-WT/pMiR-TYMS-Mut). HEK293 cells were co-transfected with pMiR-TUG1-WT or pMiR-TUG1-Mut and miR-197-3p mimics or negative control (NC). A Renilla luciferase-expressing plasmid pRL-TK (Promega) used as control was also co-transfected. Cells were harvested and luciferase activity was determined using the Dual Luciferase Reporter Assay Kit (Promega) at 24 h after transfection. The results are expressed as relative luciferase activity (firefly luciferase/Renilla luciferase).

### 2.10. Statistical analysis

All statistical analyses were performed using SPSS 22.0 software (IBM). Data are expressed as the Mean ± SD for at least three separate experiments. The Student's t-test, χ^2^ test or Fisher's exact test was used for comparisons between groups. Overall survival was calculated by Kaplan-Meier survival analysis and compared using the log-rank test. Two-sided *P*-values were calculated, and a probability level of 0.05 was chosen for statistical significance.

## 3. Results

### 3.1. LncRNA TUG1 is up-regulated in 5-Fu resistant CRC cell lines and tissues and correlates with poor prognosis

**Figure 1:**
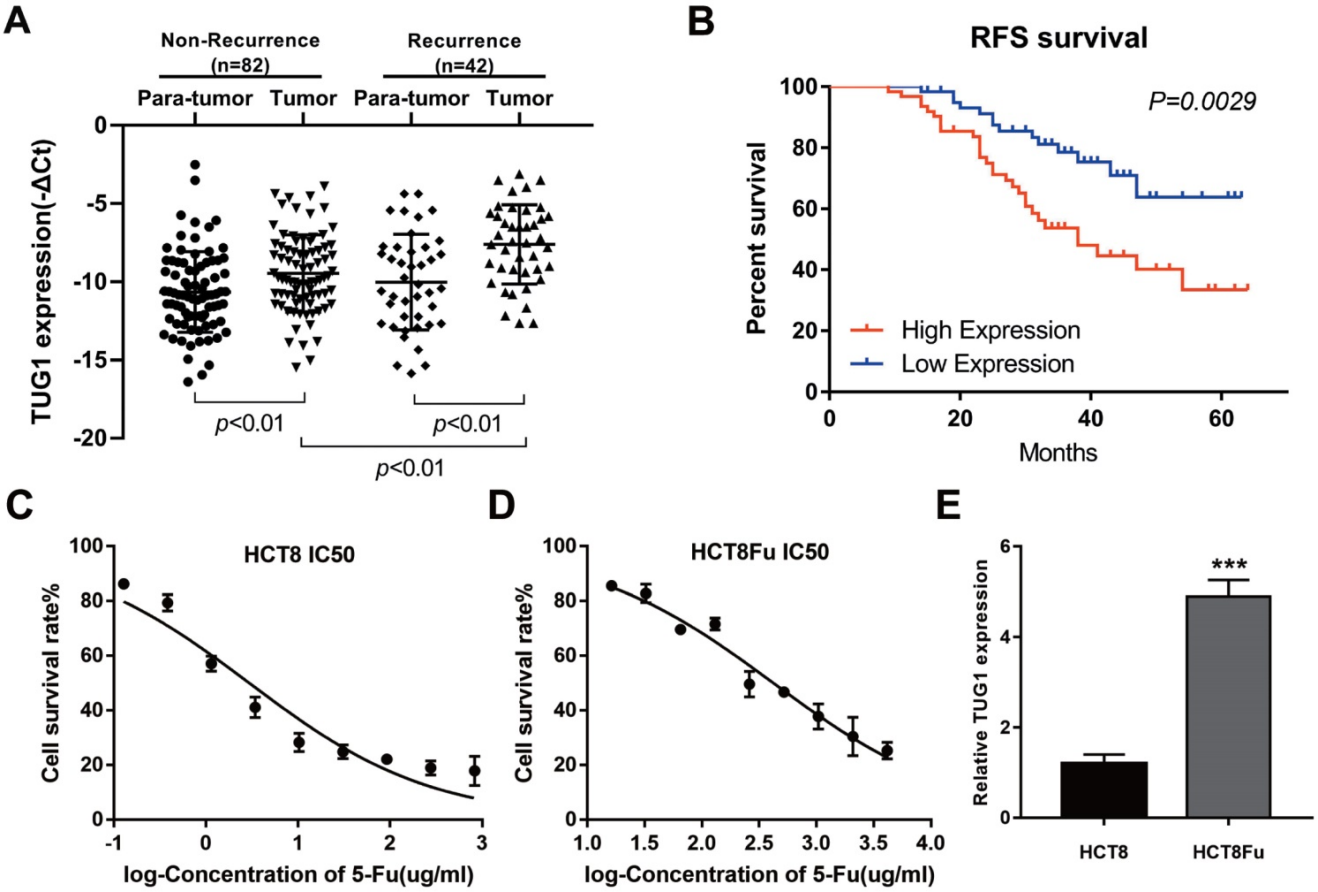
** Elevated lncRNA TUG1 was expressed in CRC tissues and cell lines.** (A) Quantitative RT-PCR (qRT-PCR) analysis of TUG1 expression in CRC tissues and pair-matched adjacent normal samples (Non-recurrence group n = 82, and Recurrence group n = 42). **P < 0.01 *versus* adjacent normal samples group or non-recurrence group. (B) Kaplan-Meier analyses of the associations between TUG1 expression level and recurrence-free survival of patients with CRC (the log-rank test was used to calculate *P*-values). **P = 0.0029 < 0.01 *versus* low TUG1 expression group. (C, D) The cytotoxic effect of 5-Fu on the HCT8 and HCT8Fu cells was measured by MTT assay and IC50 value was calculated (422.17 ± 1.84 μg/ml for HCT8Fu cells and 12.95 ± 2.64 μg/ml for HCT8 cells). (E) qRT-PCR analysis of TUG1 expression in 5-Fu resistant HCT8Fu cell line and parental sensitive HCT8 cell line. ***P < 0.001 *versus* HCT8 cell line. Data are presented as mean ± SD from three independent experiments.

LncRNA TUG1 expression levels were examined in CRC tissues that were divided in the case without recurrence (N=82) and case with recurrence group (N=42). The clinical parameters of CRC patients in this study are presented in Table 1. As shown in Fig. 1A, TUG1 was significantly up-regulated in tumor tissues resected from the case with recurrent CRC patients and low in the case without recurrence. We further examined whether TUG1 expression correlated with CRC prognosis. Kaplan-Meier survival analysis indicated that high TUG1 expression in CRC tissues was significantly associated with a lower rate of disease progression (Fig. 1B). To further understand the significance of TUG1 overexpression in CRC, we set out to identify the potential associations between TUG1 expression and patients' clinicopathological features. The detailed relationships between the TUG1 expression status and clinicopathological variables of 124 patients are shown in Table 2. Noticeably, high expression of TUG1 in CRC had a significant correlation with the depth of tumor and AJCC stage. The 50% inhibitory concentration (IC50) value was much higher in HCT8Fu cells compared with the HCT8 cells (422.17±1.84 μg/ml vs. 12.95±2.64 μg/ml) (Fig. 1C and D). Then, we detected the expression levels of TUG1 in 5-Fu resistant HCT8Fu cell line and its parental 5-Fu sensitive cell line HCT8. The results indicated that TUG1 expression level in 5-Fu resistant HCT8Fu cells were significantly increased compared with 5-Fu sensitive cells (Fig. 1E). These results demonstrated that the lncRNA TUG1 was possibly involved in the occurrence of CRC recurrence and may serve as a biomarker to predict the chemoresponse and prognosis of CRC patients.

### 3.2. TUG1 promotes 5-Fu resistance in CRC cells

**Figure 2:**
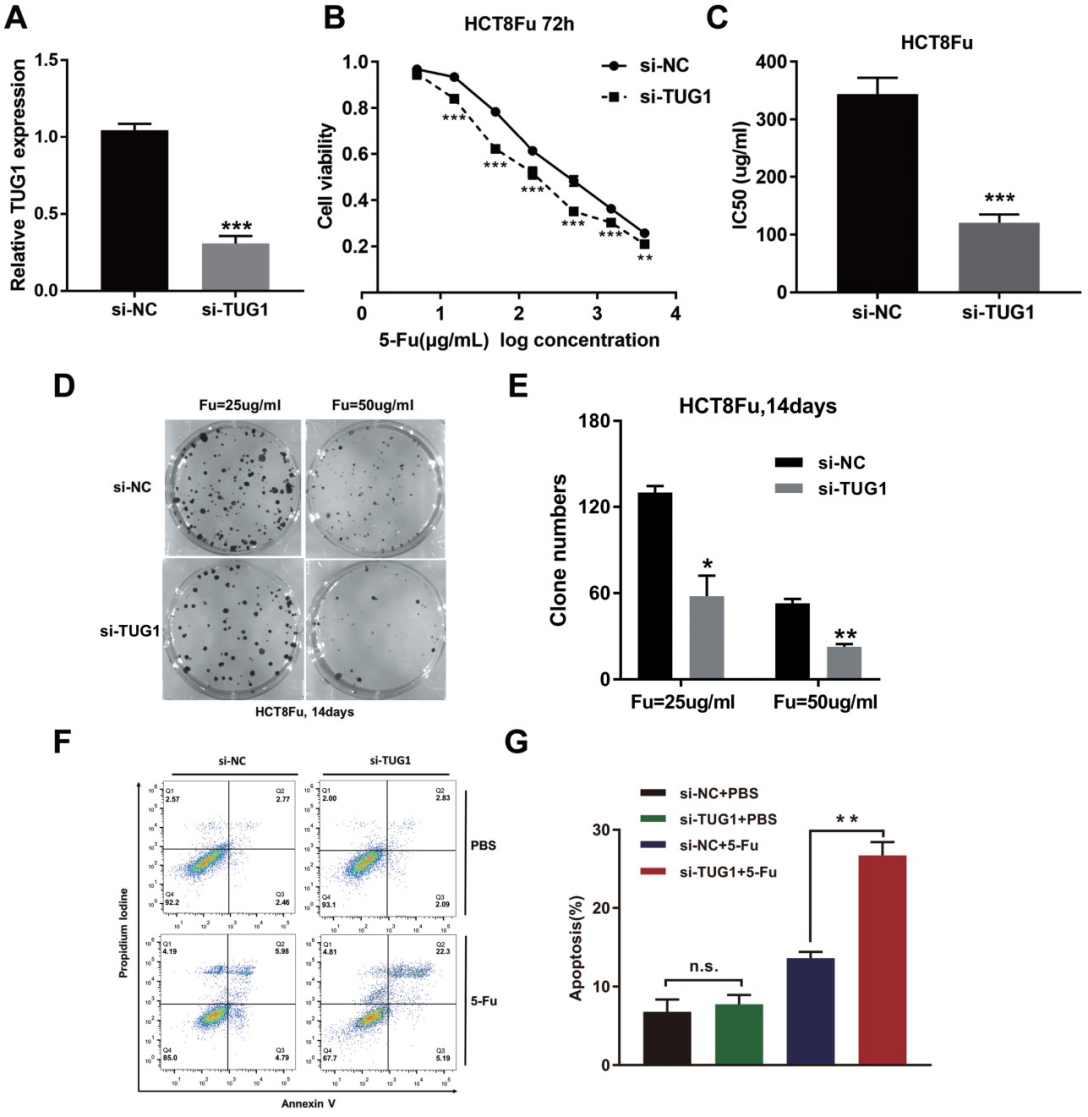
** TUG1 promotes 5-Fu resistance in CRC cells.** (A) Validation of knockdown efficacy of TUG1 in CRC cell line by qRT-PCR. ***P < 0.001 *versus* si-NC transfected group. (B) The cell sensitivity of HCT8Fu cells transfected with si-NC or si-TUG1 to 5-Fu was evaluated using the MTT assay upon exposure to the step-up concentration of 5-Fu for 72 h. **P < 0.01 and ***P < 0.001 *versus* si-NC transfected group. (C) The IC50 value of cells in the si-NC or si-TUG1 group was calculated. ***P < 0.001 *versus* si-NC transfected group. (D, E) The cell proliferation in response to anticancer drugs was examined using colony formation analysis, exposed to 25μg/ml or 50μg/ml 5-Fu for two weeks. Columns are the average of three independent experiments. *P < 0.05 and **P < 0.01 *versus* si-NC transfected group. (F, G) The percentage of apoptotic cells was determined by flow cytometric analysis. Columns are the average of three independent experiments. **P < 0.01 *versus* si-NC transfected group. Data are presented as mean ± SD from three independent experiments.

To investigate the biological functions of lncRNA TUG1 in the chemoresistance of CRC against 5-Fu, we performed MTT assay and colony formation analysis to examine the effect of TUG1 on cell sensitivity and proliferation to 5-Fu anticancer drug in HCT8Fu cells. HCT8Fu cells were transfected TUG1 siRNA, and control siRNA served as a negative control (NC) (Fig. 2A). MTT assay suggested that TUG1 knockdown significantly enhanced HCT8Fu cells sensitive to 5-Fu (Fig. 2B). IC50 value of 5-Fu in response to TUG1 down-regulation was measured. Compared with HCT8Fu cells transfected with si-NC, the IC50 value of 5-Fu in cells transfected with si-TUG1 was reduced by 64.9% (Fig. 2C). Similarly, colony formation assays revealed that cell proliferation was significantly suppressed in TUG1-downregulated HCT8Fu cells compared with NC-transfected cells exposed to 25μg/ml and 50μg/ml 5-Fu for two weeks (Fig. 2D and E). Furthermore, we performed FACS analysis to quantify apoptosis via double staining of cells with annexin V-FITC and PI. The results indicated that TUG1 knockdown increased apoptosis significantly compared with cells transfected with negative siRNA after 5-Fu administration (Fig. 2F and G). These results indicate that TUG1 suppresses the sensitivity of 5-Fu in CRC cells by inducing CRC cell apoptosis.

### 3.3. TUG1 mediated 5-Fu resistance in CRC cells via suppressing miR-197-3p

**Figure 3:**
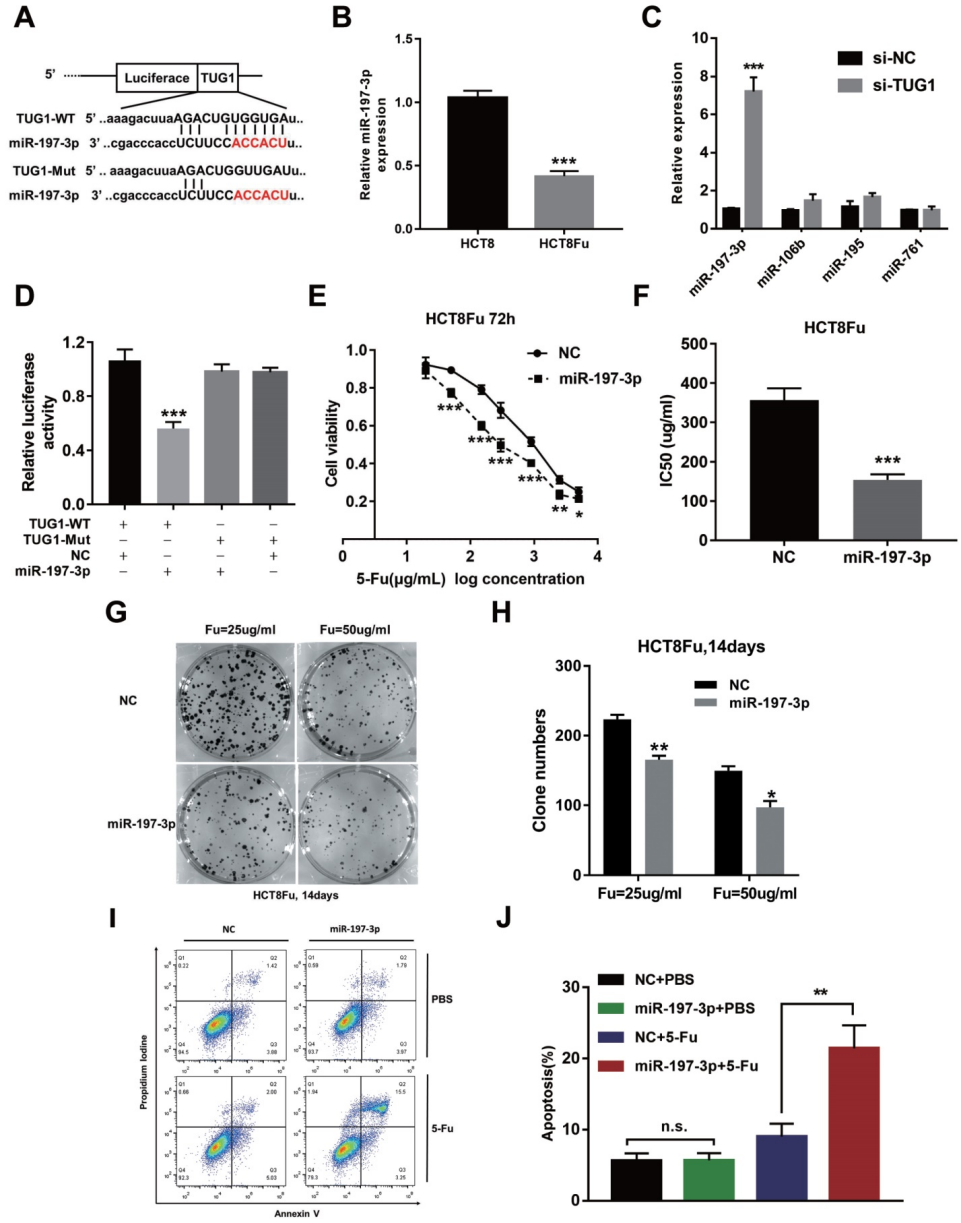
** TUG1 mediated 5-Fu resistance in CRC cells via suppressing miR-197-3p.** (A) Schematic illustration of the predicted binding sites between TUG1 and miR-197-3p, and mutation of potential miR-197-3p-binding sequence in TUG1. (B) The expression level of miR-197-3p was tested by q-PCR in HCT8 and HCT8Fu cells. ***P < 0.001 *versus* HCT8 group. (C) The expression of several drug-resistance-related microRNAs was examined by q-PCR after TUG1 knockdown. ***P < 0.001 *versus* si-NC transfected group. (D) HEK293T cells were transfected with miR-197-3p mimics and pMiR-TUG1-WT or pMiR-TUG1-mutant plasmids, and the luciferase activity was normalized by luciferase activity/Renilla activity. ***P < 0.001 *versus* pMiR-TUG1-WT and NC mimics co-transfected group. (E) The cell sensitivity of HCT8Fu cells transfected with NC mimics or miR-197-3p mimics to 5-Fu was evaluated using the MTT assay upon exposure to the step-up concentration of 5-Fu for 72 h. *P < 0.05, **P < 0.01 and ***P < 0.001 *versus* NC mimics transfected group. (F) The IC50 value of cells in the NC mimics or miR-197-3p mimics group was calculated. ***P < 0.001 *versus* NC mimics transfected group. (G, H) The cell proliferation in response to anticancer drugs was examined using colony formation analysis, exposed to 25μg/ml or 50μg/ml 5-Fu for two weeks. Columns are the average of three independent experiments. *P < 0.05 and **P < 0.01 *versus* NC mimics transfected group. (I, J) The percentage of apoptotic cells was determined by flow cytometric analysis. Columns are the average of three independent experiments. **P < 0.01 *versus* NC mimics transfected group. Data are presented as mean ± SD from three independent experiments.

Recently, emerging evidences showed that lncRNAs contained motif with complementary sequence to microRNAs (miRNAs). In an attempt to uncover whether TUG1 could interact with miRNAs, Starbase v.2.0 was used to predict potential microRNAs, which could interact with TUG1. We identified miR-197-3p may be a ceRNA that regulated by TUG1. The binding sites of miR-197-3p on TUG1 were indicated in Fig. 3A. The expression of miR-197-3p expression was decreased in HCT8Fu resistant cell line compared with sensitive cell line (Fig. 3B). We next performed q-PCR analysis for testing the expression level of several drug-resistant related microRNAs after TUG1 knockdown. Results showed that miR-197-3p was significantly up-regulated in si-TUG1 transfected cells (Fig. 3C). This data suggested that TUG1 may negatively regulate miR-197-3p. To explore whether TUG1 could interact with miR-197-3p, we constructed the luciferase reporter plasmids containing wild-type or mutant miR-197-3p putative binding sites in TUG1 (TUG1-WT and TUG1-Mut). The results of a luciferase assay demonstrated that compared to negative control (NC), co-transfection of TUG1-WT and miR-197-3p mimics resulted in significant weakening of fluorescence in HEK293T cells, but the effects vanished when we mutated the putative miR-197-3p binding sites in TUG1 (co-transfection of TUG1-Mut and miR-197-3p mimics), suggested that miR-197-3p could directly bind to TUG1 mRNA (Fig. 3D). MTT assays showed that miR-197-3p overexpression also re-sensitized HCT8Fu cells to 5-Fu (Fig. 3E and F). In addition, colony formation assays revealed that cell proliferation was significantly suppressed after miR-197-3p up-regulated (Fig. 3G and H). Extensively, FACS analysis showed that miR-197-3p increased apoptosis significantly compared with cells transfected with NC mimics after 5-Fu administration (Fig. 3I and J). Taken together, we conclude that TUG1 mediated 5-Fu resistance in CRC cells via suppressing miR-197-3p.

### 3.4. TUG1 regulated TYMS expression through miR-197-3p

**Figure 4:**
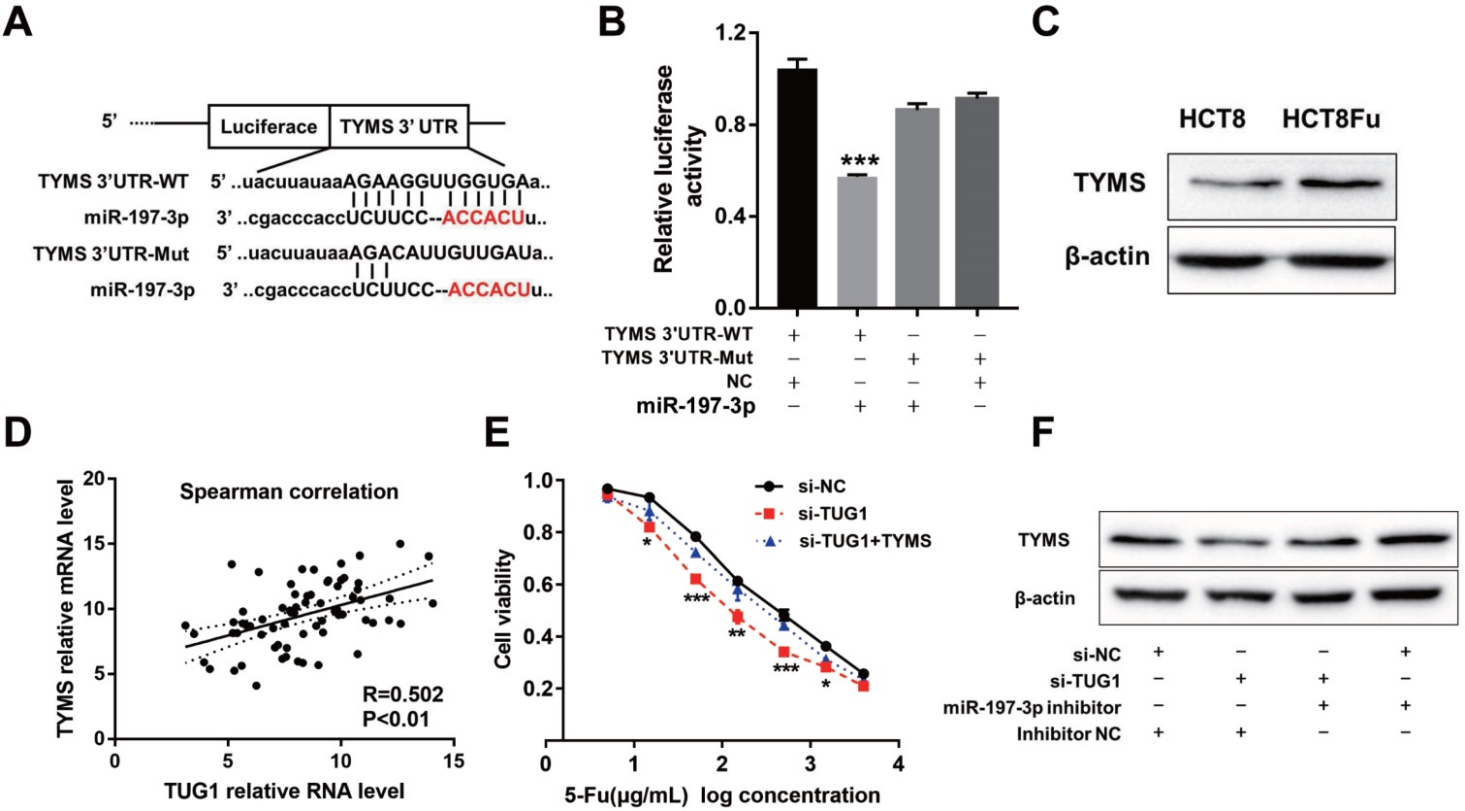
** TUG1 regulated TYMS expression through miR-197-3p.** (A) Schematic illustration of the predicted binding sites between TYMS and miR-197-3p, and mutation of potential miR-197-3p binding sequence in TYMS. (B) The relative luciferase activity in HEK293T cells with and without miR-197-3p overexpression was measured when transfected with WT or Mut luciferase plasmids. ***P < 0.001 *versus* pMiR-TYMS-WT and NC mimics co-transfected group. (C) Western blotting was used to determine the expression level of TYMS in HCT8Fu cells and their parental cells. (D) CRC tumors were subject to q-PCR for detecting the correlation between TUG1 level and TYMS level. (E) MTT cell proliferation assay was performed in HCT8Fu cells transfected with si-TUG1 or si-TUG1/TYMS and treated with the indicated concentrations of 5-Fu. *P < 0.05, **P < 0.01 and ***P < 0.001*versus* si-NC transfected group. (F) Western blotting was used to determine the TYMS expression level for si-TUG1 and miR-197-3p inhibitor co-transfected HCT8Fu cells.

Using the bioinformatics algorithms TargetScan, we identified that miR-197-3p potentially targeted TYMS. Dual-luciferase reporter assays found that miR-197-3p mimics led to the attenuation of fluorescence of the wildtype 3'-untranslated region (3'-UTR-WT), but had no effect on the mutant 3'-UTR (3'-UTR-Mut) of TYMS (Fig. 4A and B). Increased expression of TYMS was observed in 5-Fu resistant cell line compared with the parental cell line (Fig. 4C). Q-PCR analysis showed that TYMS expression level was positively associated with TUG1 in clinical recurrent tumor samples (Fig. 4D). These data indicated that TYMS was a direct target of miR-197-3p and was positively correlated with TUG1 expression and 5-Fu resistance. Next, we explored whether TYMS was the functional target of TUG1 in 5-Fu resistant CRC cells. Si-TUG1 was transfected with or without pcDNA-TYMS into HCT8Fu cells.

MTT assays revealed that the TYMS overexpression eliminated 5-Fu sensitivity caused by si-TUG1 (Fig. 4E). Significantly, results of the reversal experiment showed that while TUG1 down-regulation led to decreased expression of TYMS, simultaneous miR-197-3p down-regulation was able to reverse the inhibition of TYMS expression (Fig. 4F), indicating that the increased expression of TYMS caused by 5-Fu treatment was partly dependent on TUG1.

**Figure 5:**
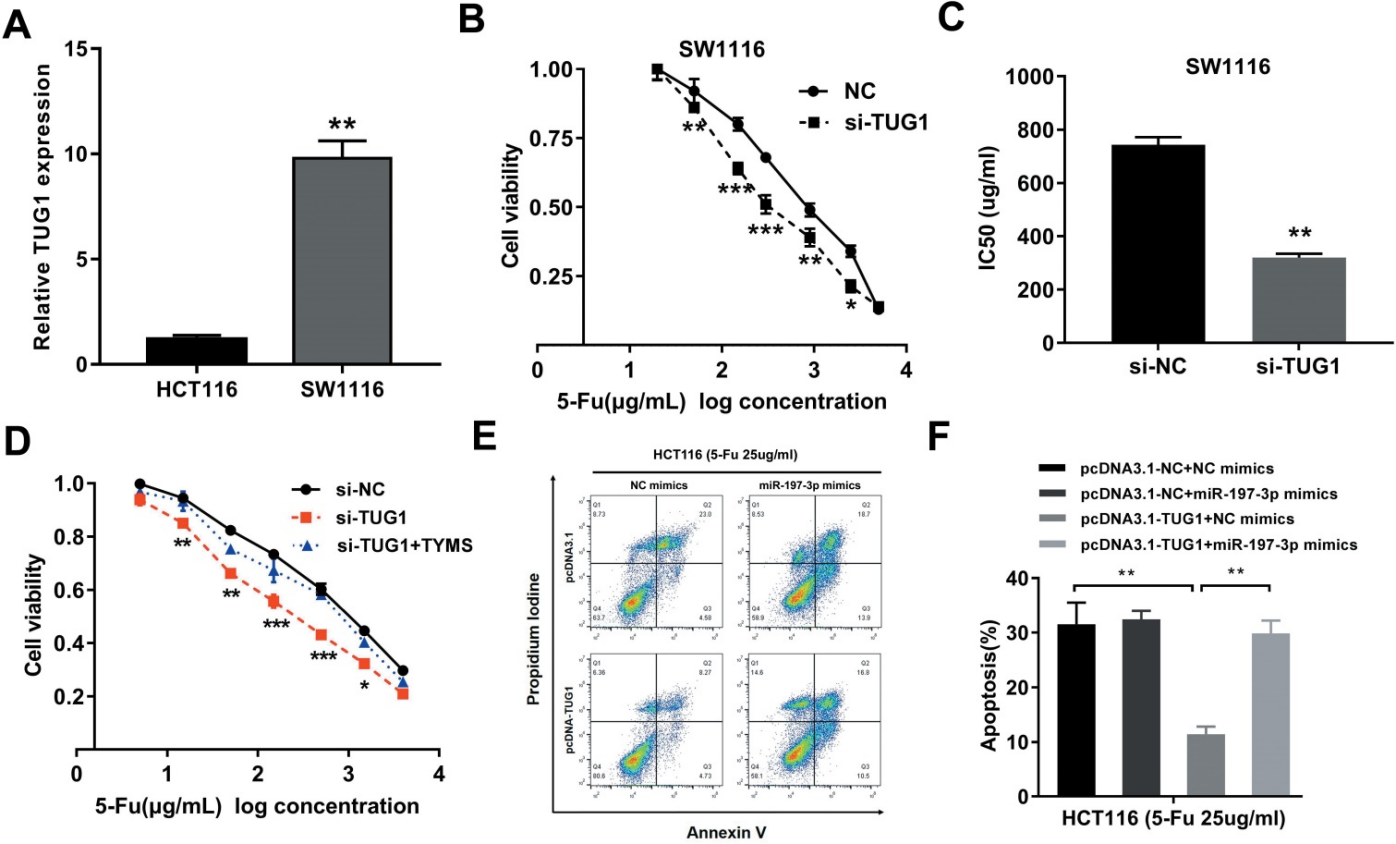
** TUG1 promotes 5-Fu resistance in primary 5-Fu resistant SW1116 cells** (A) qRT-PCR analysis of TUG1 expression in primary 5-Fu resistant SW1116 cell line and primary 5-Fu sensitive HCT116 cell line. **P < 0.01 *versus* HCT116 cell line. (B) The cell sensitivity of SW1116 cells transfected with si-NC or si-TUG1 to 5-Fu was evaluated using the MTT assay upon exposure to the step-up concentration of 5-Fu for 72 h. *P < 0.05, **P < 0.01 and ***P < 0.001 *versus* si-NC transfected group. (C) The IC50 value of cells in the si-NC or si-TUG1 group was calculated. **P < 0.01 *versus* si-NC transfected group. (D) MTT cell proliferation assay was performed in SW1116 cells transfected with si-TUG1 or si-TUG1/TYMS and treated with the indicated concentrations of 5-Fu. *P < 0.05, **P < 0.01 and ***P < 0.001 *versus* si-NC transfected group. (E, F) The percentage of apoptotic cells was determined by flow cytometric analysis. Columns are the average of three independent experiments. **P < 0.01 *versus* si-NC transfected group. Data are presented as mean ± SD from three independent experiments.

Considering that the HCT8Fu was generated in a stepwise manner by exposing drug-sensitive HCT8 cells to increasing doses of 5-Fu, cells with IC50 values above 400μg/ml were regarded as 5-Fu resistant. According to this criterion, another cell line SW1116 was identified as primary 5-Fu resistant in our previous report 20. We then used SW1116 as primary 5-Fu resistant cell line and HCT116 as primary sensitive cell line to further validate our main findings and conclusions. qRT-PCR showed that TUG1 expression was obviously up-regulated in primary 5-Fu resistant SW1116 cell line as compared with HCT116 cell line (Fig. 5A). MTT assay suggested that TUG1 knockdown significantly enhanced SW1116 cells sensitive to 5-Fu (Fig. 5B). Compared with SW1116 cells transfected with si-NC, the IC50 value of 5-Fu in cells transfected with si-TUG1 was reduced by 59.6% (Fig. 5C). These results indicated that TUG1 promotes 5-Fu resistance in primary 5-Fu resistant SW1116 cell line. Further rescue assays revealed that the TYMS overexpression eliminated 5-Fu sensitivity caused by si-TUG1 (Fig. 5D). Mechanically, TUG1 induced 5-Fu resistance was abolished by miR-197-3p in primary 5-Fu sensitive HCT116 cell line, which was confirmed by flow cytometric apoptosis analysis (Fig. 5E and 5F). Collectively, these data strongly support the hypothesis that lncRNA-TUG1 promotes CRC cells 5-Fu chemoresistance via miR-197-3p/TYMS axis.

## 4. Discussion

5-Fu is a base analogue that acts as an anti-cancer drug primarily for digestive tract tumors 21. Although 5-Fu is a basic chemotherapeutic drug for CRC, it is inefficient in most CRC patients due to drug resistance 22. Despite a great deal of efforts have been made to identify potential predictive markers of 5-Fu, there is still a need of accurate markers to discriminate patients who are likely to benefit from 5-Fu therapy 23, 24. Therefore, improving the sensitivity to drug resistance remains an urgent requirement for CRC chemotherapies. Long non-coding RNAs (lncRNAs) are emerging as new and valuable molecules and have been identified as oncogenes or tumor suppressors that are involved in tumorigenesis and chemotherapy resistance 25, 26. Tsang *et al*. reported that H19 induces MDR1-associated drug resistance in human hepatocellular carcinoma cells 27. Li *et al*. reported that snaR contributes to 5-Fu resistance in human colon cancer cells 28. Li and colleagues found that HOTTIP promotes progression and gemcitabine resistance by regulating HOXA13 in pancreatic cancer 29. Wang and colleagues found that lncRNA-LINC00161 sensitizes osteosarcoma cells to cisplatin-induced apoptosis by regulating the miR-645-IFIT2 axis 30. Among these lncRNAs, Taurine Upregulated Gene 1 (TUG1) has been reported as cancer-related, and can bind to polycomb repressive complex 2 (PRC2) or PRC1 as well as repress gene expression 31, 32. As the length of the TUG1 lncRNA is not short, ~6.7 kb, it is plausible that TUG1 has multiple functions, which remain unknown.

In our study, we firstly investigated the potential clinical value of TUG1 in the prognosis of 5-Fu treatment among CRC patients. High TUG1 expression was positively associated with disease recurrence in CRC patients receiving 5-Fu based therapies. In addition, a negative correlation was found between TUG1 expression and RFS, which indicated the prognostic value of TUG1 among CRC patients. This is also of considerable therapeutic significance because a TUG1 restoration method that may provide a new modulation strategy to overcome chemoresistance is important. Then, using gene intervention technology to knockdown TUG1, cell proliferation and resistance to 5-Fu was markedly decreased. In response to chemotherapy, cell apoptosis was one of the most commonly activated pathways, and disruption of apoptosis would facilitate multidrug resistance. 5-Fu is a pyrimidine analog, which works through irreversible inhibition of thymidylate synthase and results in apoptosis of cancer cells 33. In this manuscript, we showed that TUG1 knockdown could significantly increase apoptosis after 5-Fu treatment, indicating that TUG1 knockdown could enhance the susceptibility of CRC cells to 5-Fu via increasing 5-Fu induced apoptosis. Although there was a significant effect of TUG1 on 5-Fu resistance, investigation on more types of chemotherapy was needed to enhance the significance of TUG1 on chemoresistance.

Multiple studies have documented that lncRNAs harbor potential miRNA-responsive elements and function as competitive platforms for miRNAs in multiple types of cancer, thus reducing the repression of mRNAs 34-36. Through bioinformatics algorithms, we predicted a novel microRNA, miR-197-3p, as a potential target of lncRNA-TUG1. Dual-luciferase reporter gene assay revealed the direct interaction between TUG1 and miR-197-3p. The expression of miR-197-3p was down-regulated when the 5-Fu resistance occurs. Moreover, TUG1 knockdown significantly increased the miR-197-3p level in CRC cells. All these results suggest TUG1 works as a ceRNA to sponge and suppress miR-197-3p. Our data also showed that miR-197-3p could re-sensitize the sensitivity of 5-Fu to CRC cells and induce more apoptosis after 5-Fu treatment. These results also demonstrated that miR-197-3p might work as a tumor suppressor in CRC. Our future investigation will explore the roles of miR-197-3p in CRC progression.

Thymidylate synthase (TYMS) is one of key enzymes of the 5-Fu catabolic pathway, and this enzyme has been associated with the response to 5-Fu based therapy. Studies showed that CRC patients with a low expression of TYMS were more sensitive to fluoropyrimidine-based chemotherapy 37. The present study also demonstrated that the expression of TYMS was increased in the 5-Fu resistant cell line compared with sensitive cells. The bioinformatic analysis was performed to find that miR-197-3p contained binding sites for TYMS 3'UTR. The direct binding was further validated by dual-luciferase reporter assay. To clarify whether miR-197-3p was involved in the TUG1-mediated expression of TYMS, the combinations of transfections were conducted, and the results confirmed that TUG1 knockdown combined with miR-197-3p inhibition most rescued the expression level of TYMS, suggesting that TYMS could have an important role in TUG1-mediated 5-Fu resistance. Besides, our results showed that the expression of TUG1 was positively correlated with that of TYMS in CRC patients' samples. We then investigated whether TYMS was the direct downstream target of TUG1. The results indicated that although knockdown of TUG1 re-sensitized the resistant cells to 5-Fu, overexpression of TYMS largely reversed the effects that knockdown of TUG1 exerted. Therefore, it would highlight the significance that TUG1 induced 5-Fu resistance mainly by inhibiting miR-197-3p/TYMS axis in CRC.

In summary, the present study for the first time revealed that TUG1 was upregulated in CRC recurrence tissues and 5-Fu resistant cell lines. Knockdown of TUG1 significantly re-sensitized resistant cells to 5-Fu exposure as well as induced cell apoptosis. TUG1 influenced 5-Fu resistance though directly binding to the miR-197-3p and affected the expression of its target gene TYMS. Our findings highlight the potential value of TUG1 as a predictive tool for assessing the response to 5-Fu treatment and also suggest that the inhibition of TUG1 may be a useful therapeutic strategy to reverse the resistance to 5-Fu.

## Supplementary Material

Supplementary tables.

## Figures and Tables

**Table 1:** Characteristic of 124 patients with colorectal cancer.

Characteristics	Case without recurrence	Case with recurrence	p-value
	n=82	n=42	
Age (years)			
<60	40	20	0.903
≥60	42	22	
Gender			
Male	47	21	0.438
Female	35	21	
Tumor size			
<5cm	26	18	0.219
≥5cm	56	24	
Location			
Colon	35	20	0.601
Rectum	47	22	
Differentiation			
Well/Moderate	72	36	0.742
Poor	10	6	
Depth of tumor			
T1+T2	34	21	0.365
T3+T4	48	21	
AJCC stage			
Ⅱ	39	11	0.022
Ⅲ	43	31	
TUG1			
Low	48	14	0.008
High	34	28	

**Table 2:** Correlation of expression of TUG1 with clinicopathologic features.

Characteristics	TUG1		*p-*value
	Low	High	
Age (years)			
<60	30	30	1.000
≥60	32	32	
Gender			
Male	37	31	0.279
Female	25	31	
Tumor size			
<5cm	22	22	1.000
≥5cm	40	40	
Location			
Colon	28	27	0.857
Rectum	34	35	
Differentiation			
Well/Moderate	53	55	0.592
Poor	9	7	
Depth of tumor			
T1+T2	33	22	0.047
T3+T4	29	40	
AJCC stage			
Ⅱ	33	17	0.003
Ⅲ	29	45	
