# Supplementary material for: Long non-coding RNA TUG1 mediates 5-fluorouracil resistance by acting as a ceRNA of miR-197-3p in colorectal cancer
Source: J Cancer. 2019 Aug 7;10(19):4603–13. doi: 10.7150/jca.32065 (PMC6746119; doi:10.7150/jca.32065)
Supplement: Supplementary file 1 — Supplementary tables. [file jcav10p4603s1.pdf]

Supporting Tables

Table S1 The primers (mRNA) of real-time PCR

Name	Forward primer(5'→3')	Reverse primer(5'→3')
β-actin	GTCAACGGATTTGGTCTGTATT	AGTCTTCTGGGTGGCAGTGAT
TYMS	TCTGGAAGGGTGTTTTGGAG	CCTCCACTGGAAGCCATAAA
TUG1	CCAGACCCTCAGTGCAAACT	CAATCAGGAGGCACAGGAC

Table S2 The primers (miRNA) of real-time PCR

MiRNA	Primer Sequence(5'→3')
miR-197-3p	TTCACCACCTTCTCCACCCAGC
miR-195	TAGCAGCACAGAAATATTGGC
miR-761	ACAGCAGGCACAGACCAC
miR-106b	AGCCACATCGCTCAGA
U6	CAAGGATGACACGCAAATTCG

Table S3 The sequence of microRNA

Name	Sequence
miR-197-3p mimics	5’-UUCACCACCUUCUCCACCCAGC-3’<br>5’-UGGGUGGAGAAGGUGGUGAAUU-3’
Negative control mimics	5’-UUCUCCGAACGUGUCACGUTT-3’<br>5’-ACGUGACACGUUCGG AGAATT-3’
miR-197-3p inhibitor	5’-GCUGGGUGGAGAAGGUGGUGGUGAA-3’
Negative control Inhibitor	5’-CAGUACUUUUGUGUAGUCAA-3’

Table S4 SiRNA sequence

Name	Sense (5’-3’)	Antisense (5’-3’)
TUG1 SiRNA	CAGUCCUGGUGAUUUAGAC AGUCTT	GACUGUCUAAAUCACCAGGAC UGTT
